# Discharge Disposition After Limb Amputation in a Publicly Funded Health Care System

**DOI:** 10.1177/00469580231176354

**Published:** 2023-05-29

**Authors:** Samuel Kwaku Essien, Audrey Zucker-Levin

**Affiliations:** 1 University of Saskatchewan, Saskatoon, SK, Canada

**Keywords:** amputation, discharge disposition, income status, comorbidity, epidemiology

## Abstract

Understanding discharge disposition (DD) after limb amputation (LA) surgery allows health care providers and policy makers to adapt resources based on need. Studying independent prognostic factors for DD after LA in Canada eliminates the significant influence of payor source, as reported by researchers in the United States. We hypothesize disparities exist among DDs after LA in a publicly funded health care system. Retrospective review of Saskatchewan’s linked administrative health data from 2006 to 2019 was used to identify independent socio-demographic factors, amputation levels, amputation predisposing factors (APF), and surgical specialty on 5 DD’s: inpatient, continuing care, home with support services (H/W), home with no support services (H/WO), and those who died at the hospital after LA. We found age, amputation level, and APF play a significant role in determining discharge to all dispositions; gender was significantly associated with discharge to continuing care and H/WO; place of residence was associated with discharge to inpatient facilities, continuing care, and H/W; income was not associated with any DD other than H/W; surgical specialty was associated with discharge to all dispositions except death. The findings suggest that disparities in DD following LA exist even after eliminating the influence of payor source. Health care providers and policy makers should consider these findings in preparation for future needs.

**What do we already know about this topic?**
Discharge disposition after limb amputation is often influenced by the patient’s ability to afford care or payor source.
**How does your research contribute to the field?**
This study identifies disparities in discharge disposition after limb amputation exist without the influence of payor source.
**What are your research’s implications toward theory, practice, or policy?**
Our findings inform health care providers with insight into disparities and give policy makers and health care administrators data to apprize policy and address disparities.

## Introduction

Discharge disposition (DD) after limb amputation (LA) surgery is determined based on multiple criteria considering available resources with the ultimate goal of maximizing mobility and function.^1-3^ Patients may discharge home after LA if they are mobile with or without an assistive device (eg, wheelchair), in stable health, can participate in daily life activities independently or with an established social support system, and are able to access needed rehabilitation services.^4^ Discharge to a post-acute care facility (PACF) such as an inpatient rehabilitation facility (IRF), skilled nursing facility (SNF), or long-term care facility (LTC) may be indicated to optimize independence, and physical function, to provide ongoing medical management or to assist with daily living needs.^4^ Discharge disposition after LA may directly or indirectly be influenced not only by health care needs but by personal (eg, age, gender, and residence), economic (eg, income status), and co-morbid factors, as well as level of LA (eg, partial foot vs above knee) and specialty of the surgeon who performed the LA.^5-11^

To complicate matters, in the United States, there is evidence that DD is influenced by payor source, with adults covered by Medicaid benefits more likely to discharge home or to a SNF than to an IRF after traumatic spinal cord injury, major trauma,^12^ or burn injuries compared to non-Medicaid patients or patients covered by commercial insurance.^13-15^ Similarly, patients with low income were more likely to be discharged home after major LA compared to those discharged to an SNF.^5^

Studying DD after LA in Canada eliminates the influence of payor source as Canada’s provincially managed publicly funded health care system is designed to cover “medically necessary health care services on the basis of need, rather than the ability to pay.”^16^ This system provides universal and equitable care to Canadians. In contrast, private healthcare, more common than public coverage^17^ in the US, touted as more efficient, accountable, and perhaps more effective, establishes disparity with little evidence of improved health outcomes.^18^ Further, causes of LA in Canada are similar to that of other countries, with dysvascular disease as the leading cause, followed by trauma, neoplasm, and congenital anomalies.^19^ Saskatchewan is among the Canadian provinces ranked highest in LA incidence (6-year rate of 28.3 per 100 000 vs 22.9 Canadian average).^19^ And socioeconomic disparities are known to negatively impact indigenous Canadians who experience LA at a higher rate than non-indigenous Canadian residing in Saskatchewan (153.9 ± 17.3 [SD] vs 31.1 ± 2.3 [SD] per 100 000 respectively).^20^

Examining DD after LA without the complication of payor source will inform health care providers and policy makers on factors that influence health care utilization and if disparities exist among patient cohorts. Therefore, the objective of this study was to describe the pattern of DD after LA by demographic factors, amputation predisposing factors (APF), and levels of amputation.

## Methods

### Data

Discharge disposition, levels of amputation, and demographic and APF were examined using linked administrative health data from the Province of Saskatchewan, Canada, between January 1, 2006, and December 31, 2019. The Saskatchewan healthcare system is covered under the provincially managed Canadian universal single-payer healthcare system, and its health services utilization is documented in the administrative health databases housed in Saskatchewan Health Quality Council. The three linked datasets used for this study include the *Person Health Registration System* (PHRS), which contains demographic factors and health coverage information; *Discharge Abstracts Database* (DAD) containing acute-care hospital information; and the *Physician Characteristics and Mobility file* containing physician specialty and mobility information. All 3 databases were linked electronically with encrypted unique identification numbers. The implementation of ICD-10-CA/CCI classifications was adopted across other provinces in Canada in 2006^21-23^; hence the selection of this date allows for study results to be more applicable to other jurisdictions in Canada. This epidemiologic study of DD did not follow a study protocol.

Saskatchewan residents discharged from a hospital after LA procedure were identified in the DAD using the procedure codes stipulated in the Canadian Classification of Health Intervention (CCI) (1SN93, 1SQ93, 1TA93, 1TK93, 1TM93, 1TV93, 1VA93, 1VC93, 1VG93, 1VQ93, 1UB93, 1UE93, 1UF93, 1UG93, 1UH93, 1UI93, 1UJ93, 1UK93, 1UM93, 1WA93, 1WE93, 1WI93, 1WJ93, 1WK93, 1WL93, 1WM93, 1WN93).^24^ The description of each code has been reported elsewhere.^25^

### Discharge Disposition Descriptors

The main study outcome, discharge disposition, was limited to 5 groups: inpatient institutions (eg, IRF and other acute, sub-acute, acute psychiatric, acute cancer center, acute pediatric center), continuing care (eg, SNF), home or settings with support services (H/W) (eg, skilled care services such as daily dressing changes, wound care, physical therapy, and or occupational therapy) and these include facilities such as senior’s lodge, attendant care, home care and supportive housing, home with no support service (H/WO), and those who died at the hospital after LA. The small sample size precluded further assessment of other DDs (eg, detained by social services, addiction treatment center, palliative care facility/hospice, and cadaveric donor admitted for organ/tissue retrieval).

### Other Variables

A broad range of socio-demographic factors, amputation levels, APF, and surgical specialty were considered in the current study. Socio-demographic factors were categorized as follows: age (0-49, 50-59, 60-69, and 70+ years), gender (female/male), place of residence (urban/non-urban), and income quintile (low-income quintile, middle-high quintile, and highest quintile). Place of residence was defined by postal code population (non-urban < 1000 and urban > 1000)^26,27^ with the years 2018 to 2019 being a proxy of the place of residence in 2017.

Amputation levels were identified based on the CCI codes with a minor LA group consisting of individuals with “only finger/hand” and “only toe/part foot” and through or proximal to the wrist/ankle as major amputation levels.^28^ Patients with two or more different LA levels (eg, 2/more different minor levels or 2/more major levels, or a combination of both minor and major levels) were all categorized as “Other” since further breakdown would have resulted in small cell size, which violates the collaboration agreement with the data trustees. Also, due to a proportion of patients with “only finger/hand” and “only toe/part foot,” this minor LA group was separated from those with multiple different levels to specifically assess how that was associated with discharge to the various dispositions. Surgical specialties included general, vascular, orthopedic, and others (eg, family practice/general practice medicine).

We also explored the pattern of 6 of the most common APF using ICD-10-CA diagnostic codes for diabetes (eg, E10-E14), PVD (eg, I70, I72-I78, and I80-I99), trauma (eg, S480-S481, S680-S684, S980-S984, and T050-T059), cancer (eg, C400- C403, C408-C409, C436-C437, C764-C765, and D036-D037), congenital (eg, Q710-Q731, Q738, Q740-Q742, Q748-Q749), and infection (eg, A498, A499, L08, L98, A40, B95, B96, and B99). The small sample size of less than 6 when individually stratified by DD precluded independent evaluation of the APF’s of trauma, cancer, congenital, and infection; thus, they were combined into the “Other” category.

### Analysis

The distribution of DD by socio-demographic factors, APF, and amputation level, in Saskatchewan, was described using numbers (N) and frequencies (%). Further, a Chi-square test of equality of proportions^29,30^ was carried out to determine whether factor levels in each of the 5 DDs are the same or differ from others. In addition, pairwise proportion multiple comparison tests^31^ were performed where multiplicity adjustments were accounted for using the Bonferroni adjustment method. Factor levels were statistically significantly different from each other at *P* < .05. All pairwise analyses were carried out using the pairwise.prop.test function in R-software.^32^

## Results

Table 1 illustrates the DD and demographic characteristics of patients who met the study’s inclusion criteria for APF and 5 DD after LA in Saskatchewan. Over the 14-year period, a total of 4312 LA procedures met the study inclusion criteria, of which 1856 (43%) were discharged to H/WO, 1002 (23.2%) were discharged to an inpatient facility, 886 (20.6%) were discharged to H/W, 408 (9.5%) discharged to continuing care, and 160 (3.7%) died. The majority, 3109 (72.1%), of people who had LA were male, and 2668 (61.9%) lived in an urban setting. Most, 1903 (44.1%), LA occurred in people 50 to 69 years of age, followed by 1540 (35.7%) in those 70+ years, with 869 (20.2%) in the youngest age group, 0 to 49 years. Most LA, 2549 (59.1%) were minor. Diabetes was the most frequent APF, with 3068 (71.2%) cases, followed by PVD without diabetes, 696 (16.1%) and 548 (12.7%) other. Income was inversely proportional to the number of cases, with 2338 (54.2%) in the lowest income quintile followed by the middle-income quintile, 1426 (33.1%) and 548 (12.7%) in the highest income quintile. General surgeons performed the majority of procedures, followed by vascular surgeons, orthopedic surgeons, and other surgeons, 1590 (36.9%), 1479 (34.3%), 677 (15.7%), and 566 (13.1%), respectively.

**Table 1.** The Study Patient’s Characteristics (N = 4312).

N = 4312		Distribution of discharged disposition expressed as a proportion of the overall sample (N) of levels of each factor/variable				
Variables/factors	N (%)	Inpatient 1002 (23.2)	Continuing care 408 (9.5)	Home with support 886 (20.6)	Home with no support 1856 (43)	Died 160 (3.7)
Gender						
Female	1203 (27.9)	283 (23.5)	154 (12.8)	230 (19.1)	483 (40.2)	53 (4.4)
Male	3109 (72.1)	719 (23.1)	254 (8.2)	656 (21.1)	1373 (44.2)	107 (3.4)
*P*-value^+^		*P* = .781	***P* < .001**	*P* = .149	***P*** **=** **.017**	*P* = .133
Residence						
Non-urban	1644 (38.1)	494 (30.1)	90 (5.4)	284 (17.3)	726 (44.2)	50 (3.0)
Urban	2668 (61.9)	508 (19.0)	318 (11.9)	602 (22.6)	1130 (42.4)	110 (4.1)
*P*-value^+^		***P* < .001**	***P* < .001**	***P* < .001**	*P* = .245	*P* = .068
Age/years						
0-49	869 (20.2)	125 (14.4)	29 (3.3)	175 (20.2)	527 (60.6)	13 (1.5)
50-69	1903 (44.1)	387 (20.3)	135 (7.1)	472 (24.8)	853 (44.8)	56 (3.0)
70+	1540 (35.7)	490 (31.8)	244 (15.8)	239 (15.5)	476 (30.9)	91 (6.0)
*P*-value^+^		***P* < .001**	***P* < .001**	***P* < .001**	***P* < .001**	***P* < .001**
Amputation level						
Minor*	2549 (59.1)	363 (14.2)	54 (2.1)	679 (26.6)	1416 (55.6)	37 (1.5)
Major	1550 (36.0)	579 (37.4)	327 (21.1)	166 (10.7)	375 (24.2)	103 (6.6)
Other	213 (4.9)	60 (28.2)	27 (12.7)	41 (19.3)	65 (30.5)	20 (9.3)
*P*-value^+^		***P* < .001**	***P* < .001**	***P* < .001**	***P* < .001**	***P* < .001**
Amputation predisposing factors (APF)						
Diabetes	3068 (71.2)	769 (25.1)	302 (9.8)	705 (23.0)	1169 (38.1)	123 (4.0)
PVD	696 (16.1)	179 (25.7)	94 (13.5)	126 (18.1)	268 (38.5)	29 (4.2)
Other	548 (12.7)	54 (9.9)	12 (2.2)	55 (10.0)	419 (76.5)	8 (1.4)
*P*-value^+^		***P* < .001**	***P* < .001**	***P* < .001**	***P* < .001**	***P* = .011**
Income quintiles						
Highest	548 (12.7)	148 (27.0)	48 (8.8)	87 (15.9)	244 (44.5)	21(3.8)
Middle	1426 (33.1)	336 (23.6)	150 (10.5)	268 (18.8)	618 (43.3)	54 (3.8)
Low	2338 (54.2)	518 (22.2)	210 (9.0)	531 (22.7)	994 (42.5)	85 (3.6)
*P*-value^+^		*P* = .050	*P* = .246	***P* < .001**	*P* = .668	*P* = .960
Surgical specialty						
General	1590 (36.9)	358 (22.5)	138 (8.7)	357 (22.4)	666 (41.9)	71 (4.5)
Orthopedic	677 (15.7)	147 (21.7)	91 (13.4)	98 (14.5)	321 (47.4)	20 (3.0)
Vascular	1479 (34.3)	453 (30.6)	171 (11.6)	356 (24.0)	445 (30.1)	54 (3.7)
Other	566 (13.1)	44 (7.8)	8 (1.4)	75 (13.2)	424 (74.9)	15 (2.7)
*P*-value^+^		***P* < .001**	***P* < .001**	***P* < .001**	***P* < .001**	*P* = .144

* Minor-consists of only finger/hand and toe/part foot and Other LA level category-consists of other minor LA levels, and those with both minor and major levels.

+ Unadjusted *P*-value. (*P* < .05)

### Within-Disposition Differences in Proportions of Patients by Gender

The distribution (%) of DD stratified by patient’s gender is summarized in Table 1. Differences (*P* < .001) in the proportion of gender distribution existed in 2 DDs, with more females (12.8%) than males (8.2%) discharged to continuing care and more males (44.2%) than females (40.2%) discharged to H/WO. No gender differences were identified for discharge to an inpatient facility (*P* = .781), H/W (*P* = .149), or those who died at the hospital (*P* = .133).

### Within-Disposition Differences in Proportions of Patients by Place of Residence

The distribution (%) of DD stratified by place of residence is depicted in Table 1. Place of residence was significantly (*P* < .001) associated with discharge to an inpatient facility, H/W, and continuing care between non-urban and urban dwellers. More non-urban residents (30.1%) were discharged to inpatient facilities than urban residents (19.0%), and more urban residents were discharged to H/W (22.6%) or to continuing care (11.9%) when compared to their non-urban counterparts (17.3% and 5.4% respectively). Residence type was not associated with discharge to H/WO (44.2% vs 42.4%, *P* = .245) or those who died at the hospital after LA (3.0% vs 4.1% *P* = .068).

### Within-Disposition Differences in Proportions of Patients by Age

The Chi-Square analyses represented in Table 1 show that age plays a significant role in determining discharge to all dispositions after LA (*P* < .001). The multiple comparison results in Table 2 further identified significant differences among all age groups within each DD, except for patients who died in the hospital after LA, where DD did not differ among those aged 0 to 49 and 50+ years (*P* = .098).

**Table 2.** Within-Disposition Differences in Proportions of Patient by Age, Amputation Level, and APF.

Disposition	Age					Amputation level					APF				
	Group	%	Multiple comparison of groups		*P*-value^+^	Group	%	Multiple comparison of groups		*P*- value^+^	Group	%	Multiple comparison of groups		*P*-value^+^
Inpatient	0-49	14.4	0-49	50-69	.001	Minor	14.2	Minor	Major	<2e-16	Diabetes	25.1	Diabetes	PVD	.999*
	50-69	20.3	0-49	70+	<2e-16	Major	37.4	Minor	Other	3.1e-7	PVD	25.7	Diabetes	Other	2.4e-14
	70+	31.8	50-69	70+	6.1e-14	Other	28.2	Major	Other	.033	Other	9.9	PVD	Other	5.5e-12
Continuing care	0-49	3.3	0-49	50-69	<.001	Minor	2.1	Minor	Major	<2e-16	Diabetes	9.8	Diabetes	PVD	.017
	50-69	7.1	0-49	70+	<2e-16	Major	21.1	Minor	Other	<2e-16	PVD	13.5	Diabetes	Other	2.3e-8
	70+	15.8	50-69	70+	1.6e-15	Other	12.7	Major	Other	.016	Other	2.2	PVD	Other	8e-12
H/W support service	0-49	20.2	0-49	50-69	.024	Minor	26.6	Minor	Major	<2e-16	Diabetes	23.0	Diabetes	PVD	.018
	50-69	24.8	0-49	70+	.014	Major	10.7	Minor	Other	.068*	PVD	18.1	Diabetes	Other	3.3e-11
	70+	15.5	50-69	70+	9e-11	Other	19.3	Major	Other	.001	Other	10.0	PVD	Other	<.001
H/WO support service	0-49	60.6	0-49	50-69	4.5e-14	Minor	55.6	Minor	Major	<2e-16	Diabetes	38.1	Diabetes	PVD	.990*
	50-69	44.8	0-49	70+	<2e-16	Major	24.2	Minor	Other	9.7e-12	PVD	38.5	Diabetes	Other	<2e-16
	70+	30.9	50-69	70+	3e-16	Other	30.5	Major	Other	.170*	Other	76.5	PVD	Other	<2e-16
Died	0-49	1.5	0-49	50-69	.098*	Minor	1.5	Minor	Major	<2e-16	Diabetes	4.0	Diabetes	PVD	.996*
	50-69	3.0	0-49	70+	1.6e-6	Major	6.6	Minor	Other	1.1e-13	PVD	4.2	Diabetes	Other	.015
	70+	6.0	50-69	70+	8.2e-5	Other	9.3	Major	Other	.550*	Other	1.4	PVD	Other	.026

* Indicate factors levels are not significantly different from each other (*P* < .05).

+ All *P*-values adjusted using the Bonferroni adjustment method.

The frequency of patients discharged to an inpatient facility or continuing care and those who died in hospital after LA increases as age increases. The rate of admission to an inpatient facility was 31.8% for people 70+ years of age, 20.3% for people 50 to 69 years of age, and 14.4% for people 0 to 49 years of age. Likewise, the rate of admission to a continuing care facility was 15.8% for people 70+ years of age, 7.1% for people 50 to 69 years of age, and 3.3% for people 0 to 49 years of age. People 70+ years of age died at a rate of 6.0%, while those 50 to 69 and 0 to 49 died at a rate of 3.0% and 1.5%, respectively. The frequency of patients discharged H/WO decreased as age increased (age 70+ years: 30.9%; age 50-69 years: 44.8%; age 0-49 years: 60.6%). Finally, patients aged 50 to 69 years were more likely to discharge H/W (24.8%) than their younger (20.2%) or older (15.5%) counterparts. Likewise, patients aged 0 to 49 years were more likely to discharge home with support services (20.2%) than their older counterparts aged 70+ years (15.5%).

### Within-Disposition Differences in Proportions of Patients by Amputation Level

The Chi-Square analyses in Table 1 show that amputation level plays a significant role in determining discharge to all dispositions after LA (*P* < .001). The multiple comparison results in Table 2 further identified significant differences among all levels of LA within each DD except for H/WO or those who died.

The rate of admission to an inpatient facility was significantly higher after major LA (37.4%) when compared to both “other” levels of LA (28.2%) and minor LA (14.2%) and significantly higher when “other” levels of LA are compared to minor LA. Similar differences are identified for discharge to continuing care, with 21.1% after major LA, 12.7% after “other” LA, and 2.1% after minor LA. The frequency of patients discharged H/W was higher after minor LA (26.6%) when compared to those discharged home with major LA (10.7%) or after “other” LA (19.3%) and significantly higher when “other” LA is compared to major LA. However, the difference in the frequency of minor LA discharge H/W compared to “other” levels of LA was not significant (19.3% vs 26.6%, *P* = .068). Patients with minor LA were more likely to discharge H/WO (55.6%) than after major LA (24.2%) or “other” levels of LA (30.5%). Likewise, patients with minor LA were less likely to die in the hospital (1.5%) when compared to their counterparts who had major LA (6.6%) or “other” levels of LA (9.3%). No differences in the frequency of discharge H/WO (24.2% vs 30.5%, *P* = .170) or death (6.6% vs 9.3%, *P* = .550) in the hospital were identified when major LA was compared to “other” levels of LA. (It seems the font size is different for the entire paragraph. please check and correct this)

### Within-Disposition Differences in Proportions of Patients by APF

The Chi-Square analyses represented in Table 1 show that APF plays a significant role in determining discharge to all dispositions after LA (*P* < .001). The multiple comparison results in Table 2 further identify significant differences among APF’s within each DD, except for inpatient facility, H/WO, and those who died in the hospital after LA. Thus, patients who were discharged to inpatient, H/WO, and those who died in the hospital did not differ by diabetes or PVD status but differed in “other” APF (*P* > .05). Patients with diabetes and patients with PVD were discharged to inpatient facilities at a higher rate (25.1% and 25.7%, respectively) than patients with “other” APF (9.9%). Significant differences were found among all APFs for discharge to continuing care and H/W. Patients with PVD were discharged to continuing care more frequently (13.5%) than patients with diabetes (9.8%) and patients with “other” APF (2.2%). Also, patients with diabetes were more likely to discharge to continuing care than patients with “other” APF. Patients with diabetes were discharged H/W at a higher rate (23.0%) than patients with PVD (18.1%) or patients identified as “other” (10.0%). Also, patients with PVD were more likely to receive support services at home (18.1%) than patients with “other” APF (10.0%). Patients with APF identified as “other” were discharged H/WO more frequently (76.5%) than patients with diabetes or PVD (38.1% and 38.5%, respectively). Patients with APF identified as “other” were less likely (1.4%) to die in the hospital after LA than patients with diabetes (4.0%) or PVD (4.2%).

### Within-Disposition Differences in Proportions of Patients by Income Levels

The distribution (%) of DD stratified by income level is summarized in Table 1. No income differences were identified for discharge to an inpatient facility (*P* = .050), continuing care (*P* = .246), H/WO (*P* = .668), or those who died at the hospital (*P* = .960). The Chi-square results show income played a role in discharge to H/W (*P* < .001); however, income was not associated with discharge to any of the other dispositions, including death. The multiple comparison results in Table 3 further identify that discharge to H/W is not different in patients of high-income status when compared to patients of middle-income status (15.9% vs 18.8%, respectively; *P* = .444); however, patients of low income (22.7%) are more likely to discharge H/W than patients of high or middle-income status.

**Table 3.** Within-Disposition Differences in Proportions of Patient by Surgical Specialty and Income Level.

Disposition	Surgical specialty						Income level			
	Group	%	Multiple comparison of groups		*P*- value^+^		Group	%	Multiple comparison of groups	*P*- value^+^
Inpatient	General	22.5	General	Orthopedic	.998*					
	Orthopedic	21.7	General	Vascular	2.6e-6					
	Vascular	30.6	General	Other	1.0e-13					
	Other	7.8	Orthopedic	Vascular	<.001					
			Orthopedic	Other	1.2e-10					
			Vascular	Other	<2e-16					
Continuing care	General	8.7	General	Orthopedic	.005					
	Orthopedic	13.4	General	Vascular	.057*					
	Vascular	11.6	General	Other	3.7e-08					
	Other	1.4	Orthopedic	Vascular	.990*					
			Orthopedic	Other	8.5e-14					
			Vascular	Other	4.2e-12					
H/W support service	General	22.4	General	Orthopedic	<.001	High	15.9	High	Middle	.444*
	Orthopedic	14.5	General	Vascular	.994*	Middle	18.8	High	Low	.002
	Vascular	24.0	General	Other	2.1e-05	Low	22.7	Middle	Low	.015
	Other	13.2	Orthopedic	Vascular	3.2e-06					
			Orthopedic	Other	.999*					
			Vascular	Other	6.7e-07					
H/WO support service	General	41.9	General	Orthopedic	.100*					
	Orthopedic	47.4	General	Vascular	8.4e-11					
	Vascular	30.1	General	Other	<2e-16					
	Other	74.9	Orthopedic	Vascular	5.4e-14					
			Orthopedic	Other	<2e-16					
			Vascular	Other	<2e-16					
Died	General	4.5	General	Orthopedic	.710*					
	Orthopedic	3.0	General	Vascular	.999*					
	Vascular	3.7	General	Other	.460*					
	Other	2.7	Orthopedic	Vascular	.989*					
			Orthopedic	Other	.990*					
			Vascular	Other	.995*					

* Indicate factors levels are not significantly different from each other (*P* < .05).

+ All *P*-values adjusted using the Bonferroni adjustment method.

### Within-Disposition Differences in Proportions of Patients by Surgical Specialty

The distribution (%) of DD stratified by surgical specialty is summarized in Table 1. Except for death proportions which did not differ among surgeon specialties (*P* = .144), the association of surgeon specialty was statistically significant (*P* < .001) in the discharge of patients to the four destinations (inpatient facility, continuing care, H/W, and H/WO). The multiple comparison results in Table 3 further identified significant differences among all surgical specialties within each DD.

Vascular surgeons discharged patients to inpatient facilities at a higher rate (30.6%) than all other surgical specialties: general surgeons (22.5%), orthopedic surgeons (21.7%), and “other” surgeons (7.8%). The rate of discharge to inpatient facilities was similar for general surgeons and orthopedic surgeons (22.5% vs 21.7%, *P* = .998) and significantly less for “other” surgeons (22.5% vs 7.8%, *P* = 1.0e-13).

Discharge to continuing care did not differ between general surgeons (8.7%) and vascular surgeons (11.6%) or between orthopedic surgeons (13.4%) and vascular surgeons. Orthopedic surgeons discharged patients to continuing care more frequently than general surgeons, and “other” surgeons (1.4%) discharged patients to continuing care less frequently than all other surgical specialties.

A comparable proportion of patients who had LA surgery by general and vascular surgeons were discharged H/W (22.4% and 24.0%, respectively). Likewise, orthopedic surgeons and “other” surgeons were similar (13.2% and 14.5%, respectively) when discharging patients to H/W. General and vascular surgeons discharged patients to H/W more frequently than orthopedic and other surgeons.

Finally, “other” surgeons discharged their patients to H/WO more frequently (74.9%) than all other surgical specialties (Orthopedic 47.4%, General 41.9%, and Vascular 30.1%). Vascular surgeons discharged their patients to H/WO less frequently than orthopedic surgeons and general surgeons.

## Discussion

Studying DD after LA in Canada is valuable as it can identify health care utilization and disparities without the influence of payor sources. This investigation analyzed data spanning a period of 14 years (2006-2019), which is uncommon in this field of research, and found that almost two-thirds (63.6%) of patients after LA in Saskatchewan were discharged home (H/W and H/WO). Of those discharged home, the majority were discharged H/WO (43.0%) with 20.6.% discharged to H/W. Fewer patients, 32.7%, were discharged to a facility; specifically, 23.2% were discharged to an inpatient facility, and 9.5% were discharged to continuing care. Finally, 3.7% of our cohort died in the hospital. Few recent publications are available for comparison to our cohort as payor source is a major consideration for DD after hospitalization. The majority of publications regarding DD after LA are from the US and are more than 10 years old,^33^ thus do not reflect recent changes in health care reimbursement, specifically the effect of the Affordable Care Act (ACA), which was enacted in 2013 in the United States.^34^ Further, published reports on DD focus their cohort on major LA due to vascular disease.^33,35^ Although our cohort included all APF, the vast majority (87.3%) were associated with dysvascular disease (diabetes:71.2%; PVD:16.1%) with more support provided in all domains (inpatient, continued care, H/W) than those with other APF. Unfortunately, but not unexpectedly, people with dysvascular disease were more likely to die in the hospital after LA than those with other APFs. To compare our findings to previously published reports, DD after major LA was as follows: 34.9% were discharged home, 37.4% were discharged to an inpatient facility, and 21.1% were discharged to continuing care. These proportions are similar to the recent report of O’Banion et al,^35^ who identified 39% discharged to home; 27% discharged to an acute rehab facility (inpatient), and 23% discharged to SNF after lower extremity amputation due to dysvascular disease, however, they differ from those reported 20 years ago by Dillingham et al^36^ where 49.8% of patients were discharged home; 9.6% were discharged to an IRF, and 37.4% were discharged to SNF. Perhaps this trend toward more discharge to rehab facilities and fewer discharges directly home or to SNF reflects the availability of care. For example, discharging home after LA may require increased skilled services such as nursing, physical therapy, and occupational therapy, which may not be available, especially in rural and remote areas.^37^ We were not surprised that age played a significant role in all DDs. Our finding that frequency of patients discharged to an inpatient facility or continuing care and those who died in hospital after LA increases as age increases are supported by other studies. O’Banion and Lavery both found that patients who were discharged home after LA were significantly younger than those who were discharged to inpatient facilities.^35,38^ This trend is consistent with other surgical interventions, including after long bone fractures Van Der & Freburger.^39, ^40^^ We also found that patients 50 to 69 years of age were more likely to discharge H/W than their younger or older counterparts.

We found that the income quintile did not dictate DD for inpatient care, continued care, or discharge H/WO, nor was it associated with mortality while in the hospital. Interestingly, we found people of lower SES receive more home support services than their higher-income counterparts. This is similar to the findings of Kurtz Landy et al,^41^ who identified socioeconomically disadvantaged women living in Ontario, Canada, were more likely to accept public health nurse home visits during the first 4 post-partum weeks than their socioeconomically advantaged counterparts. However, the literature from the US counters our findings. Multiple reports identify people of lower socioeconomic status (SES) receive less services and were discharged to rehabilitation facilities and nursing facilities less frequently than their more affluent counterparts after hip fracture^40^; likewise, people of lower SES receive less home services than their higher-income counterparts after surgical revascularization for peripheral arterial disease.^42^ These findings support our hypothesis that studying DD after LA in Canada is valuable as it eliminates the influence of payor sources.

Our finding that females are discharged to continuing care more frequently than males after LA is supported in the literature^33^ and may be because the proportion of females living alone in Saskatchewan is greater^43^ than that of males; therefore, discharged to continuing care is needed for assistance with non-medical needs. Likewise, our finding that non-urban dwellers were discharged to inpatient facilities at a greater rate than urban dwellers and urban dwellers discharging H/W more than non-urban dwellers may be due to inadequate home care services available in non-urban areas needed for post-LA medical management.

Finally, death in the hospital did not differ among surgical specialties, but DD differed among all destinations, with patients under the care of vascular surgeons more frequently discharged to inpatient facilities followed by general and orthopedic surgeons. This was not surprising as patient demographics differ among surgical specialties, with trauma patients often managed by orthopedic surgeons who require emergent or unplanned LA, while patients with dysvascular disease, who may be in poor entry-level health, are often scheduled for procedures and managed by vascular surgeons.^44^ Planned surgical intervention gives the patient and medical team the ability to better plan DD; for example, discharging H/W may be established prior to planned surgery. Further, a more granular analysis is needed to determine if an interaction exists among surgical specialty, levels of amputation, age, and residence.

## Strengths and Limitations

There has been limited research on the use of care services and DD after limb amputation. Hence it was a strength that this study addressed this neglected but important area of post-amputation health services and present relevant findings to inform health policy. Due to data limitations, this study did not consider race, level of education, or discharge education, each of which has been identified as a significant factor in determining DD or readiness to discharge in patients after surgical intervention in other jurisdictions.^44,45^ The collective agreement between the researchers and the data trustees precluded the reporting of small numbers, especially in subgroups, to ensure confidentiality,^46^ hence limiting the study only to include 5 DD groups. Patients discharged to other dispositions and reported in the database as “left against medical advice with or without sign-out,” “detained by social services,” “addiction treatment center,” “palliative care facility/hospice,” and “cadaveric donor admitted for organ/tissue retrieval” were excluded. In addition, it is not clear if previous limb salvaging interventions had been performed or if DD changed for those discharged home or to continuing care after LA.

## Conclusion

Examining DD after LA in Saskatchewan, Canada, allowed us a better understanding of health resource utilization and to identify disparities independent of the financial burden of health care. Fourteen years of data identified that DD was not negatively associated with income, with additional support provided to lowest-income patients who were discharged home after LA in Saskatchewan. As expected, patients with major LA and dysvascular disease were discharged to inpatient and continuing care facilities at a greater rate than their counterparts with minor and “other” LA and diabetes and “other” APF. Interestingly, patients who reside in non-urban areas were more likely to discharge to inpatient facilities and less likely to go to continuing care or home with support than their counterparts in the urban area. This may be due to a lack of services available in non-urban areas. Females were more likely to discharge to continuing care and less likely to go to H/WO than males, and surgical specialty was associated with DD evidenced by the finding that vascular surgeons send the majority of their patients to inpatient care settings while other surgical specialties discharge their patients to H/WO. The findings suggest that disparities in DD following LA are substantial even after eliminating the influence of payor source. It is not clear if APF played a role in this trend. Further research is needed to determine the underlying cause of the identified disparities.
